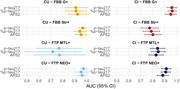# Differential Performance of *p*‐tau217 in Reflecting Amyloid and Tau Stages in Cognitively Unimpaired and Impaired Individuals

**DOI:** 10.1002/alz70856_106209

**Published:** 2026-01-08

**Authors:** Han‐Kyeol Kim, Jae Hoon Lee, Joong‐Hyun Chun, Mina Park, You Jin Kim, Tim West, Kristopher M. Kirmess, Philip B. Verghese, Daniel Connell, Joel B. Braunstein, Young Hoon Ryu, Chul Hyoung Lyoo, Hanna Cho

**Affiliations:** ^1^ Wonju Severance Christian Hospital, Yonsei University Wonju College of Medicine, Wonju, Gangwon‐do, Korea, Republic of (South); ^2^ Gangnam Severance Hospital, Yonsei University College of Medicine, Seoul, Korea, Republic of (South); ^3^ Severance Hospital, Yonsei University College of Medicine, Seoul, Korea, Republic of (South); ^4^ Gangnam Severance Hospital, Yonsei University College of Medicin, Seoul, Korea, Republic of (South); ^5^ C2N Diagnostics, LLC, Saint Louis, MO, USA; ^6^ C2N Diagnostics, LLC, St. Louis, MO, USA

## Abstract

**Background:**

This study aimed to assess the differential predictive performance of plasma *p*‐tau217 for early and advanced stages of amyloid and tau pathology in cognitively unimpaired (CU) and cognitively impaired (CI) individuals, evaluating its stage‐specific utility across cognitive states.

**Methods:**

A total of 237 participants underwent ^18^F‐florbetaben (FBB) and ^18^F‐flortaucipir (FTP) PET imaging, as well as plasma biomarker assessments, including *p*‐tau217, %p‐tau217, and APS2. Participants were categorized as CU or CI based on neuropsychological evaluations. Amyloid pathology stages were defined as FBB G+ (early amyloid pathology) and FBB Str+ (advanced amyloid pathology), while tau pathology stages were classified as FTP MTL+ (early tau pathology) and FTP NEO+ (advanced tau pathology). The predictive performance of *p*‐tau217 for each stage was assessed using ROC analysis.

**Results:**

In the CU group, plasma biomarkers demonstrated excellent predictive performance for both early (FBB G+) and advanced (FBB Str+) stages of amyloid pathology, with AUC values exceeding 0.9. Specifically, for FBB G+, *p*‐tau217 achieved an AUC of 0.954, while for FBB Str+, it reached 0.968. Regarding tau pathology, *p*‐tau217 showed high accuracy for both FTP MTL+ (AUC = 0.944) and FTP NEO+ (AUC = 0.975) in the CU group.

In the CI group, while the predictive performance for early amyloid pathology (FBB G+) remained strong (AUC = 0.961), it declined for advanced amyloid pathology (FBB Str+, AUC = 0.775). For tau pathology, *p*‐tau217 performed better for FTP NEO+ (AUC = 0.915) compared to FTP MTL+ (AUC = 0.842).

Notably, comparative analysis revealed that *p*‐tau217 reflected amyloid pathology significantly better in the CU group compared to the CI group, particularly for FBB Str+ (*p* < 0.001). Conversely, in the CI group, *p*‐tau217 showed a stronger association with advanced tau pathology (FTP NEO+) compared to early tau pathology (FTP MTL+, *p* =  0.028).

**Conclusion:**

This differential performance suggests that *p*‐tau217 more accurately reflects amyloid burden in CU individuals, while being more indicative of advanced tau pathology in CI individuals. These results highlight the stage‐ and cognitive status‐dependent utility of plasma *p*‐tau217 as a biomarker in Alzheimer's disease pathology.